# Dyskinesia in Parkinson's Disease Therapy

**DOI:** 10.1155/2012/639080

**Published:** 2012-12-13

**Authors:** Anna Rosa Carta, Andrea Giuffrida, Gilberto Fisone

**Affiliations:** ^1^Department of Biomedical Sciences, University of Cagliari, Via Ospedale 72, 09124 Cagliari, Italy; ^2^Department of Pharmacology, University of Texas Health Science Center at San Antonio, 7703 Floyd Curl Drive, San Antonio, TX 78229, USA; ^3^Department of Neuroscience, Karolinska Institutet, Retzius väg 8, 171 77 Stockholm, Sweden

L-DOPA is still regarded as the standard pharmacotherapy for the treatment of the motor symptoms of Parkinson's disease (PD). However, the efficacy of this drug is limited by the emergence of dystonic and choreic involuntary movements, generally referred to as dyskinesia. Current interventions to treat dyskinesia are mainly based on continuous delivery of L-DOPA, administration of glutamatergic drugs (i.e., amantadine), replacement or combined administration of L-DOPA with less dyskinetic, albeit less effective, dopaminergic agonists, and deep-brain stimulation of discrete regions of the basal ganglia.

Preclinical research, on the other hand, is searching for novel approaches to the treatment of L-DOPA-induced dyskinesia, targeting alternative neurotransmitter systems, such as the serotonin, adenosine, and opioid systems, or identifying abnormalities in intracellular signal transduction and synaptic plasticity associated to this condition. This Special Issue discusses recent breakthroughs in this direction and, at the same time, provides an update of the clinical features and management of L-DOPA-induced dyskinesia.

The article by J. Guridi et al. describes the mechanisms underlying the various forms of dyskinesia produced by prolonged administration of L-DOPA. Basic pathophysiological mechanisms and current treatments are presented in detail and critically discussed.

I. Aviles-Olmos et al. focus on the comparison between L-DOPA-induced dyskinesia and graft-induced dyskinesia, an analogous condition observed in response to transplantation of fetal dopaminergic cells in PD patients. This article also provides a timely discussion of the use of gene therapy in PD and of its effects on dyskinesia.

The article by N. Tambasco et al. presents the clinical and epidemiological characteristics of dyskinesia and describes the management of this condition, based on the use of various types of dopaminergic agonists. On the same line, J. C. P. Piedad and A. E. Cavanna discuss more in detail the use of pramipexole, an agonist at dopamine D_2_-type receptors, in the treatment of dyskinesia.

A number of signaling components potentially implicated in dyskinesia have been discovered during the last decade. In this regard, V. Ghiglieri et al. provide a comprehensive overview of the mechanisms and the possible pathological consequences of abnormalities affecting various forms of corticostriatal plasticity associated to L-DOPA-induced dyskinesia.

Recently, studies have pointed to the serotonergic system as a key player in the aberrant effects produced by L-DOPA and linked to the emergence of dyskinesia. The article by S. Navailles and P. De Deurwaerdère describes the evidence at the basis of this hypothesis and discusses the use of therapeutic approaches designed to prevent this phenomenon. On the same subject, E. Shin et al. discuss the experimental and clinical evidence implicating the serotonin system in L-DOPA- and graft-induced dyskinesia.

Drugs acting as antagonists at adenosine A_2A_ receptors have attracted considerable interest as antiparkinsonian and antidyskinetic agents. The article by M. Morelli et al. provides a critical appraisal of the potential therapeutic properties of these compounds, as determined in experimental models of PD and L-DOPA-induced dyskinesia.

Finally, G. Frazzitta et al. present an interesting study showing the beneficial effects produced on dyskinesia by intensive physiotherapeutic rehabilitation. These results are discussed with regard to similar studies performed in animal models of PD.

